# Co-evolutionary Rates of Functionally Related Yeast Genes

**Published:** 2007-02-18

**Authors:** Leonardo Mariño-Ramírez, Olivier Bodenreider, Natalie Kantz, I. King Jordan

**Affiliations:** 1 National Center for Biotechnology Information, National Institutes of Health, Bethesda, MD 20894, U.S.A; 2 National Library of Medicine, National Institutes of Health, Bethesda, MD 20894, U.S.A; 3 School of Biology, Georgia Institute of Technology, Atlanta, GA 30332, U.S.A

**Keywords:** Functional inference, Co-evolution, natural selection, genome evolution, gene ontology

## Abstract

Evolutionary knowledge is often used to facilitate computational attempts at gene function prediction. One rich source of evolutionary information is the relative rates of gene sequence divergence, and in this report we explore the connection between gene evolutionary rates and function. We performed a genome-scale evaluation of the relationship between evolutionary rates and functional annotations for the yeast *Saccharomyces cerevisiae.* Non-synonymous (*dN*) and synonymous (*dS*) substitution rates were calculated for 1,095 orthologous gene sets common to *S. cerevisiae* and six other closely related yeast species. Differences in evolutionary rates between pairs of genes (Δ*dN* & Δ*dS*) were then compared to their functional similarities (*sGO*), which were measured using Gene Ontology (GO) annotations. Substantial and statistically significant correlations were found between Δ*dN* and *sGO*, whereas there is no apparent relationship between Δ*dS* and *sGO*. These results are consistent with a mode of action for natural selection that is based on similar rates of elimination of deleterious protein coding sequence variants for functionally related genes. The connection between gene evolutionary rates and function was stronger than seen for phylogenetic profiles, which have previously been employed to inform functional inference. The co-evolution of functionally related yeast genes points to the relevance of specific function for the efficacy of natural selection and underscores the utility of gene evolutionary rates for functional predictions.

Many post-genomic research efforts are aimed at uncovering relationships among genes, and the yeast *Saccharomyces cerevisiae* has served as a model system for such investigations (Cherry et al. 1998). A particular emphasis has been placed on high-throughput experimental attempts to elucidate various kinds of interactions between pairs of genes (or proteins), such as physical protein-protein interactions (Krogan et al. 2006), synthetic lethal gene pairs (Tong et al. 2004) and regulatory interactions between transcription factors and promoters (Harbison et al. 2004). The characterization of such relationships has the potential to reveal important clues as to the function of individual genes. Perhaps even more importantly, this line of inquiry can reveal higher order relationships, which define groups of genes that function as integrated biological systems (Ideker et al. 2001).

In addition to the kinds of experimental approaches mentioned above, computational analyses have also been brought to bear on the discovery of functional relationships between genes. These include classic information transfer techniques that rely on sequence similarity searches, using BLAST (Altschul et al. 1997) for instance, as well as more recently developed techniques that seek to exploit information on the co-occurrence of genes in different organisms (Pellegrini et al. 1999). What many of these computational approaches share in common is a reliance, either implicit or explicit, on evolutionary information. Information transfer via BLAST rests on the fact that molecular evolution is a conservative process marked by the preservation of biochemical function among related genes. Phylogenetic profile methods, which evaluate patterns of gene presence and absence across sets of species, work because natural selection tends to maintain functionally related genes as coherent sets within evolutionary lineages.

In this manuscript, we report an attempt to assess the utility of an additional source of evolutionary information for functional inference, namely the relative rates of gene evolution. Our approach is based on a growing body of literature that points to the connections between various phenotypic aspects of genes and their rates of evolution (Wall et al. 2005; Wolf et al. 2006). Among other findings, these studies have uncovered co-evolutionary connections between particular phenotypes and rates gene of evolution. For instance, genes that encode physically interacting proteins tend to evolve at similar rates (Fraser et al. 2002) as do genes that are co-expressed across similar tissue types (Jordan et al. 2004). It stands to reason that, as a general principle, genes with similar functional affinities should have similar (average) rates of evolution. We set out to test this notion by comparing the relative rates of evolution between orthologs, detected for *S. cerevisiae* and six closely related yeast species, with their Gene Ontology (GO) functional annotations.

1,095 sets of orthologous yeast genes were identified by using all-against-all reciprocal BLASTP searches (e^−10^) between *S. cerevisiae* and six closely related species with complete whole-genome draft sequences (Cliften et al. 2003; Kellis et al. 2003): *S. paradoxus, S. mikatae, S. kudriavzevii, S. bayanus, S. castelli and S. kluyveri.* Protein sequences of each orthologous set were aligned using ClustalW (Thompson et al. 1994), and the protein alignments were used to guide inframe alignments of the corresponding DNA protein coding sequences. For each set of 7 aligned orthologous genes, pairwise non-synonymous (*dN*) and synonymous (*dS*) substitution rates were computed between *S. cerevisiae* and each of the other six species using the modified Nei-Gojobori method (Nei and Gojobori 1986) implemented in the program PAML (Yang 1997). The resulting evolutionary distance values were used to calculate pairwise distance differences (Δ*dN* Δ*dS*) between *S. cerevisiae* genes, across each of the six between-species comparisons. Specifically, for any pair of *S. cerevisiae* genes *i* & *j*: Δ*dN_ij_* = |*dN_i_* − *dN_j_*| and Δ*dS_ij_* = |*dS_i_* − *dS_j_*|. This approach allowed us to evaluate the differences in evolutionary distances for pairs of genes over a range of phylogenetic distances from *S. cerevisiae.*

A modified version of the semantic similarity method (Lord et al. 2003) was used to quantitatively assess the functional relationships between *S. cerevisiae* genes. Functional similarity coefficients between pairs of GO biological process terms – *s*(*c_k_*, *c_p_*) – were calculated by using an information content based approach. This approach takes into account both the frequency of biological process GO terms in the Saccharomyces Genome Database (SGD – http://www.yeastgenome.org/) and the structure of the GO directed acyclic graph (DAG). The DAG was used to relate query terms by their closest parent term – i.e. the lowest common subsumer (*lcs*). For any term (*c_i_*), its information content – ln *p*(*c_i_*) – is a function of its number of occurrences normalized by the total number of occurrences of all GO biological process terms in the SGD. Term-term functional similarities were measured using the information content of the query terms – ln *p*(*c_k_*) & ln *p*(*c_p_*) – and their lowest common subsumer parent term – ln *p_lcs_*(*c_k_*, *c_p_*) (Lin, 1998):

$$s(c_{k},c_{p})=\frac{2\times \left[\text{ln}p_{lcs}(c_{k},c_{p})\right]}{\text{ln}p(c_{k})+\text{ln}p(c_{p})}$$

For any gene pair *ij*, all term-term similarity values were aggregated at the level of gene products to yield *sGO_ij_* by using the average highest similarity aggregation scheme as follows (Azuaje et al. 2005). Given *m* and *n* distinct GO terms for each gene in the pair *ij*,

$$\begin{array}{c} sGO_{ij}=\frac{1}{m+n}\times [\sum_{k}\underset{p}{\text{max}}(s(c_{k},c_{p}))\\ +\sum_{p}\underset{k}{\text{max}}(s(c_{k},c_{p}))] \end{array}$$

Thus, we were able to quantify functional similarities as well as evolutionary rate differences for all pairwise relationships among the 1,095 orthologous *S. cerevisiae* genes. We then compared function with evolutionary rate to determine whether functionally related genes have more similar evolutionary rates on average. Gene pairs were sorted in ascending order according to the pairwise distance difference (Δ*dN* & Δ*dS*), grouped into 10 bins, and average binned distance differences as well as average functional similarities (*sGO*) were calculated. For all six between-species comparisons, a clear linear trend exists between Δ*dN* and *sGO* (Figure 1), whereby Δ*dN* is negatively correlated with *sGO* (Figure 2a). Five out of the six Δ*dN-sGO* correlations are statistically significant at P < 0.01 (Figure 2b). In other words, genes that are more functionally similar tend to have smaller non-synonymous distance differences, on average, than genes with increasingly different functions. The only Δ*dN-sGO* correlation that was not significant was observed for the comparison between *S. cerevisiae* and *S. paradoxus* (Figure 2b). Among the six species we analyzed, *S. paradoxus* is the most closely related to *S. cerevisiae*; therefore, the lack of significance for this particular pair probably reflects the low resolution afforded by the small evolutionary distances between the two species. Consistent with this interpretation, the strength of the Δ*dN-sGO* negative correlation, as well as its statistical significance, tends to increase together with the distance between the species being compared (Figure 2). Δ*dS*, on the other hand, shows virtually no correlation with *sGO*. The magnitudes of the Δ*dS-sGO* correlations are uniformly lower than seen for Δ*dN*; the slopes of the trend lines are notably shallower, and the signs of the correlation coefficients and trend line slopes both fluctuate between positive and negative (Figure 1 and Figure 2).

In summary, genes with similar functions tend to have similar non-synonymous evolutionary rates, on average, while synonymous substitution rates show no such relationship with function. This is not surprising given the fact that non-synonymous substitutions, which change the encoded amino acid, have a more profound effect on protein structure and function than synonymous substitutions, which do not result in an amino acid change. Natural selection operates based on function and, at the molecular level, acts primarily to remove deleterious protein coding sequence variants. Nevertheless, the distinction between the patterns observed for Δ*dN* and Δ*dS* underscores a demonstrable connection between the particular effects of natural selection and the specific annotated function of yeast genes.

Phylogenetic profiles have also been successfully employed to guide computationally based functional inferences, under the assumption that functionally related genes will have similar patterns of presence and absence across different species. We sought to compare the relationships between phylogenetic profiles and the same GO-based semantic measure of functional similarity that we found to be related to non-synonymous evolutionary rates. The phylogenetic profiles we analyzed are binary presence (1) and absence (0) vectors over a defined set of species. Two different sources of phylogenetic profiles were used in this analysis: i-Marcotte group profiles (Pellegrini et al. 1999) and ii-COG database profiles (Tatusov et al. 2003). The Marcotte profiles were based on an evaluation of 16 species, and the similarities between profiles were scored using a log-likelihood ratio as previously described (Lee et al. 2004). The COG profiles were based on the presence and absence of orthologs among 71 species, and these profiles were compared here using Jaccard and Hamming similarity measures. As with the evolutionary rates, phylogenetic profile similarities were binned in ascending order, and average *sGO* values were compared to average profile similarities. All three comparisons yield a positive correlation between profile and functional similarity (Figure 3). In other words, genes that are functionally related tend to have more similar evolutionary histories in terms of gene gain and loss. However, the magnitude and significance of this effect was not nearly as strong as seen for the comparison between function and evolutionary rate. In fact, the Marcotte profiles did not yield a significantly positive correlation with *sGO* (Figure 3a). This may be attributable to the relative sparseness of this dataset; only ~3,000 profile comparisons over 16 species were available compared to >500,000 comparisons over 71 species for the COG data set. Indeed, COG based profiles were significantly correlated with *sGO* for the Jaccard similarity measure but not when Hamming similarities were used (Figure 3b and c). The different results observed for the Jaccard and Hamming measures reflects that fact that most binary phylogenetic profiles contain many absent (0) signals, and too many of these will dominate the Hamming measure, which simply counts all positions as similar or different. The Jaccard measure attains more sensitivity by ignoring vector positions that are scored as absent for both genes. Even in this case though, the strength of the correlation is not as great as typically observed for Δ*dN-sGO*.

We have demonstrated that functionally related yeast genes co-evolve with respect to the evolutionary rate at non-synonymous coding sequence positions. This effect is observed to be highly significant for all but the most closely related species comparison. For the data analyzed here, the correlation between function and evolutionary rate is stronger than seen for function and phylogenetic profiles. Rates of gene evolution are, for the most part, determined by the strength of purifying natural selection, which involves the removal of deleterious variants. As such, the results that we report here point to a close coupling between the particular function of a gene and the efficacy of purifying selection. The robust correlations between Δ*dN-sGO* also indicate that evolutionary rate comparisons can be used aid functional inference and prediction.

## Figures and Tables

**Figure 1:**
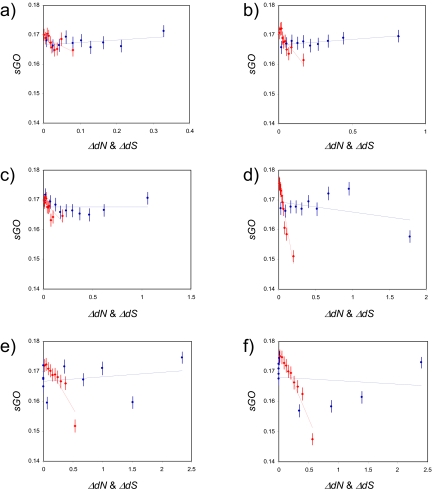
Average pairwise distance differences (x-axis) for 10 bins, with Δ*dN* shown in red and Δ*dS* shown in blue, are plotted against average pairwise GO functional similarities (*sGO* on the y-axis). The error bars correspond to 99% confidence intervals. Distances were calculated between orthologous genes of *S. cerevisiae* and a) *S. paradoxus*, b) *S. mikatae*, c) *S. kudriavzevii*, d) *S. bayanus*, e) *S. castelli*, f) *S. kluyveri*.

**Figure 2:**
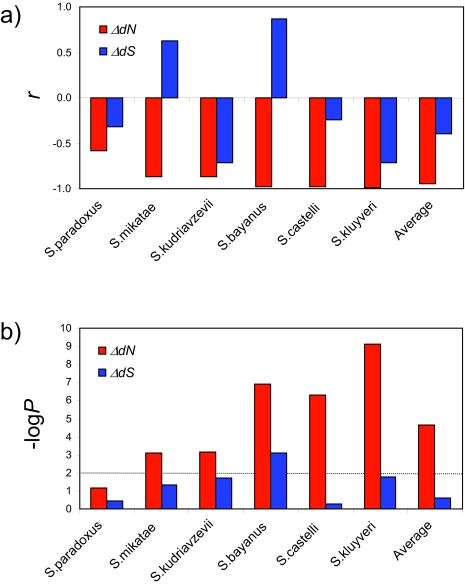
a) Pearson correlation (*r*) values are shown for the plots of distance difference (Δ*dN* Δ*dS*) vs. GO functional similarity (*sGO*) in Figure 1. b) Statistical significance (−log*P*) values are shown for the correlations in panel a. The *P* < 0.01 confidence level (−log*P* = 2) is shown. Δ*dN* related values are shown in red and Δ*dS* related values are shown in blue. Species are ordered left-to-right in terms of increasing evolutionary distance from *S. cerevisiae.*

**Figure 3:**
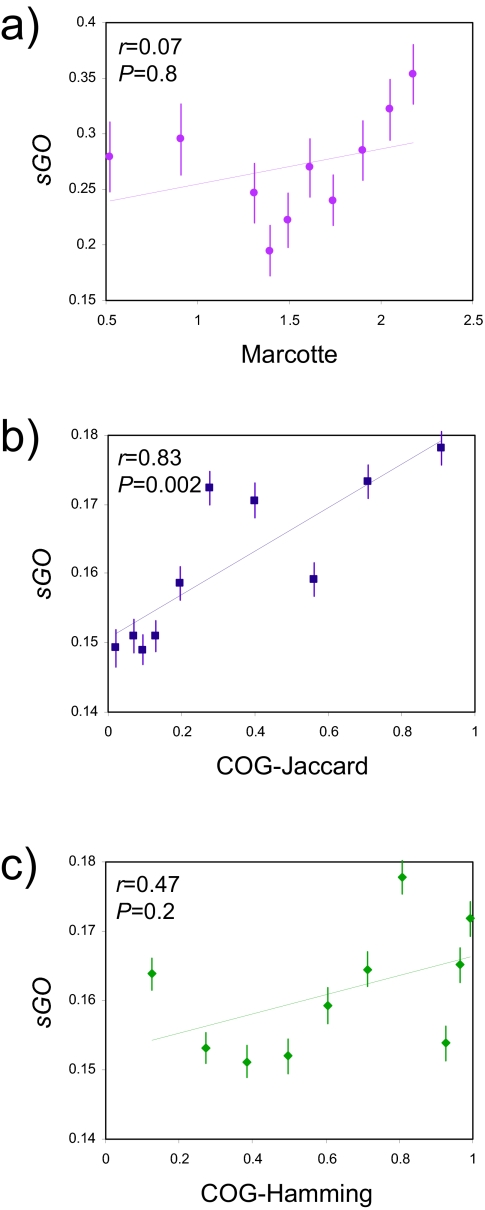
Phylogenetic profile similarity (x-axis) versus GO functional similarity (*sGO* on the y-axis). *sGO* is compared to a) Marcotte profiles, b) COG profiles evaluated via Jaccard similarity and c) COG profiles evaluated via Hamming similarity. Pearson correlation (*r*) and significance (*P*) values are shown in the inset of each plot.
